# An adaptive finite element model for steerable needles

**DOI:** 10.1007/s10237-020-01310-x

**Published:** 2020-03-09

**Authors:** Michele Terzano, Daniele Dini, Ferdinando Rodriguez y Baena, Andrea Spagnoli, Matthew Oldfield

**Affiliations:** 1 Department of Engineering and Architecture, University of Parma, Parco Area delle Scienze 181/A, 43124 Parma, Italy; 2 Department of Mechanical Engineering, Imperial College London, Exhibition Road, London, SW7 2AZ UK; 3 Department of Mechanical Engineering Sciences, University of Surrey, Guildford, Surrey GU2 7XH UK

**Keywords:** Needle insertion, Needle steering, Cohesive elements, Finite element method, Crack propagation, Programmable bevel-tip needle

## Abstract

Penetration of a flexible and steerable needle into a soft target material is a complex problem to be modelled, involving several mechanical challenges. In the present paper, an adaptive finite element algorithm is developed to simulate the penetration of a steerable needle in brain-like gelatine material, where the penetration path is not predetermined. The geometry of the needle tip induces asymmetric tractions along the tool–substrate frictional interfaces, generating a bending action on the needle in addition to combined normal and shear loading in the region where fracture takes place during penetration. The fracture process is described by a cohesive zone model, and the direction of crack propagation is determined by the distribution of strain energy density in the tissue surrounding the tip. Simulation results of deep needle penetration for a programmable bevel-tip needle design, where steering can be controlled by changing the offset between interlocked needle segments, are mainly discussed in terms of penetration force versus displacement along with a detailed description of the needle tip trajectories. It is shown that such results are strongly dependent on the relative stiffness of needle and tissue and on the tip offset. The simulated relationship between programmable bevel offset and needle curvature is found to be approximately linear, confirming empirical results derived experimentally in a previous work. The proposed model enables a detailed analysis of the tool–tissue interactions during needle penetration, providing a reliable means to optimise the design of surgical catheters and aid pre-operative planning.

**Electronic supplementary material:**

The online version of this article (10.1007/s10237-020-01310-x) contains supplementary material, which is available to authorised users.

## Introduction

Indentation and penetration of soft tissues is a common process in biology and medicine. Minimally invasive surgery by means of needle insertions is successfully applied for tumour biopsy, brachytherapy, deep brain stimulation and localised drug delivery (Elsayes et al. 2011; Lobo et al. 2012). This has significant benefits for the patient and overall cost of treatment and care; however, percutaneous needle insertions can be difficult to target and control, and surgical interventions in delicate organs need to be precise and accurate. In addition, reaching a specific location in these organs may involve following complex trajectories, instead of straight paths, for example due to no-go areas between the entry point and the target. In such cases, needle steering has the potential to correct targeting errors and steer around obstacles to reach otherwise inaccessible locations. A range of techniques has been proposed for steerable needles, including magnetic control, heating using optical fibres, external manipulation, pre-curved and nested cannulas, flexible devices with asymmetric tips (van de Berg et al. 2015). Among these, programmable bevel-tip needles (PBNs) offer unique advantages compared to other designs, notably in the context of neurosurgical drug delivery, an application area which is currently being explored in the context of a large-scale initiative funded by the European Commission (www.eden2020.eu). PBNs possess a structure with a number of interlocked segments that slide relative to one another, with motion inspired by the egg-laying channel of certain wasps. PBNs are capable of 3D steering, thanks to a bevel tip and a programmable offset between the segments (Frasson et al. 2010a; Ko et al. 2011; Ko and Rodriguez y Baena 2013), and can do so without the need for ‘duty cycling’ about the needle long axis (Swaney et al. 2013), which is particularly advantageous for highly compliant and delicate tissue traversal (Watts et al. 2019). Over the years, successive iterations of the design and manufacturing process (Frasson et al. 2010b; Leibinger et al. 2014) have resulted in the current embodiment, a medical-grade pre-commercial prototype.

Over the years, researchers have developed various theoretical and numerical models of needle penetration, which can describe the material behaviour of soft tissues and the interaction with tools having different features (Abolhassani et al. 2007; van Gerwen et al. 2012; Takabi and Tai 2017). The first attempts to develop low-resolution numerical models have considered needles as 2D beams, with external forces representing the interactions with the substrate (DiMaio and Salcudean 2003; Okamura et al. 2004). Later studies extended this approach to include the steering mechanisms of flexible needles (DiMaio and Salcudean 2005; Webster et al. 2006; Misra et al. 2010). For instance, DiMaio and Salcudean (2005) proposed an efficient algorithm, capable of describing curved trajectories in 2D, where the soft tissue is discretised by the finite element method and the needle is modelled as a thin elastic beam. A mesh adaptation was performed so that the mesh conformed to the needle steering path, and only the nodes in contact were considered, for the sake of computational efficiency.

A more thorough understanding of the interactions of flexible needles with the substrate, and of the failure mechanisms governing soft tissues, is required for the development of reliable surgical simulators (Misra et al. 2009). High-resolution finite element (FE) analyses, coupled with an accurate model of soft tissue damage, can provide a reliable means to understand, analyse and predict the processes occurring during flexible needle penetrations. Energy-based approaches and FE models have often been combined in needle insertions for fracture toughness measurement of soft tissues (Shergold and Fleck 2004; Azar and Hayward 2008; Gokgol et al. 2012), and element deletion-based methods have been used to simulate the insertion of the needle (Kong et al. 2011; Assaad et al. 2015). In addition, some authors have described damaging and cutting using the cohesive zone model (CZM) approach. Cohesive elements have been employed extensively in computational fracture mechanics, owing their fortune to the ease with which meshes are constructed, as they only need to be deployed along the path of propagation (Cornec et al. 2003; de Borst 2003). To allow for arbitrary directions of crack propagation, required in mixed-mode fracture problems, a viable approach consists in deploying cohesive elements between all continuum elements (Xu and Needleman 1994). An alternative method is based on the adaptive insertion of cohesive elements through remeshing (Camacho and Ortiz 1996; Geißler et al. 2010; Oldfield et al. 2012). Previous applications of the CZM in bevel-tip needle insertions are reported in the literature (Misra et al. 2008; Tai et al. 2013), although limited to the initial stages of indentation. Oldfield et al. (2013a) were the first to combine a detailed FE model with the cohesive zone concept, to describe the deep penetration of flexible needles in gelatine phantom tissues. Their model was capable of capturing the distinctive stages of the force–displacement curves correctly, with the penetration following a straight path defined by pre-inserted cohesive elements. The potential of this approach is that failure of the soft tissue is modelled without any reference to the specific needle geometry, or to predefined force distributions; as a consequence, it can be applied to various material behaviours and needle geometries. However, to describe steering of bevel-tip needles, crack propagation must proceed along non-predetermined paths. To date, and to the best of the authors' knowledge, the combination of a detailed FE model of needle–tissue interaction with propagation along non-predetermined paths has not been demonstrated yet.

This is not to say that much advancement has not been made in the field of modelling surgical scenarios and fracture of soft biological tissues, tool–tissue interactions and mimicking materials, such as hydrogels. On the contrary, there has been an exponential growth in the development of such models, based on emerging simulation techniques and increased computational power. A comprehensive review of the main computational techniques dealing with damage and rupture of soft tissues can be found in Gültekin and Holzapfel (2018). Among them, the extended finite element method (XFEM) has been the subject of extensive research (Agathos et al. 2016; Wan et al. 2017), with applications to the analysis of deformations and incisions in brain surgery (Vigneron et al. 2011). Meshless methods (Cheng et al. 2018) and crack phase-field approach (CPFA) (Gültekin et al. 2019), have also been used to study various aspects of damage and cutting of soft tissues and organs. Real-time simulations applied to complex interaction scenarios, such as those occurring in tool–tissue interactions, have improved considerably, thanks to new preconditioning techniques and GPU-based implementations (Courtecuisse et al. 2014). At the same time, the solution accuracy ensured by the discretisation methods remains a critical point, which has to be considered carefully (Bui et al. 2018a). A recent application of corotational CutFEM to real-time simulation of needle insertions has addressed this point specifically (Bui et al. 2018b, 2019), combining an error control method with adaptive meshing to improve the solution accuracy, while retaining the efficiency required by real-time simulations.

The purpose of the present work is to develop an algorithm that can be used for the simulation of deep insertion of needles along non-predetermined propagation paths, with a focus on accurate needle–soft material interaction. A realistic cutting model is included, with contact interactions and evolving deformation in the substrate and in the needle. Damage and fracture of the soft substrate are incorporated through cohesive elements, the stress–displacement curve of which is tuned to the results of experimental tests. A FE analysis is coupled with a modification of the mesh topology, in order to describe the increase in the length of the crack path along a non-predetermined direction. A fracture criterion is implemented to define the critical condition and the direction of propagation. The penetration path is defined through subsequent insertions of cohesive elements, based on the fracture criterion, and adaptive meshing of the surrounding region. This enabled us to model the penetration of the most complex needles, such as PBNs, where the penetration path depends on the programmable offset and is obtained as part of the solution. The results of simulations run with the PBN design are analysed in terms of the force–displacement curves and the penetration paths. A discussion is provided, where the effects of the needle's programmable offset and its stiffness are examined in relation to the simulated force–penetration curves and the resulting penetration paths.

The remainder of the paper is organised as follows: Sect. 2 focuses on the mechanics of needle insertion, with specific reference to the PBN, while Sect. 3 provides a detailed description of the iterative algorithm implemented by the authors to simulate deep needle penetration. Results are reported and discussed in Sect. 4, and conclusions are drawn to highlight the key findings of the article in Sect. 5.

## Mechanics of needle insertion

### Energetic balance of the penetration process

**Fig. 1:**
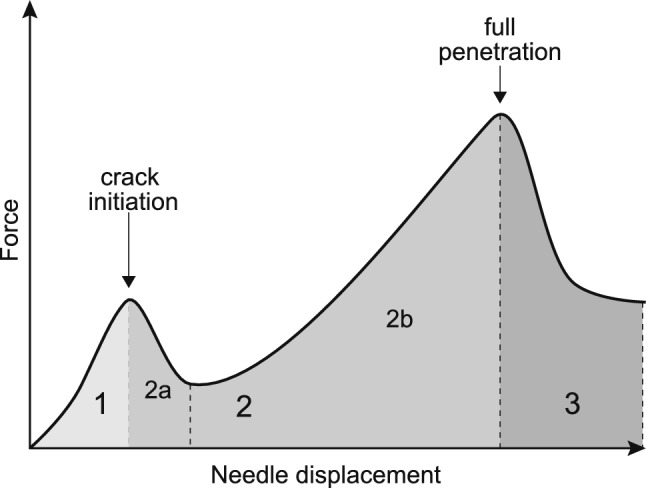
The main general stages of through-and-through needle penetration into a soft elastic solid

The mechanics of needle penetration is about the complex interaction between needle and target tissue. The detailed insertion behaviour depends on a great number of variables, e.g. the characteristics of the indenter, the properties of the target material and the penetration rate (van Gerwen et al. 2012). A flexible approach to describe penetration is based on an energetic balance of the different processes that are involved. Such a description is suitable for many diverse applications, and it has often been adopted to interpret the experimental force–displacement curves of insertion tests in different soft materials (Shergold and Fleck 2005; Azar and Hayward 2008; Spagnoli et al. 2018). The energetic balance, for an elastic material under quasi-static conditions, is summarised by the following expression:

1 $$\begin{aligned} {W_\mathrm {{ext}}} = {U_\mathrm{S}} + {U_\mathrm{f}} + U_\mathrm{G} \end{aligned}$$

where ${W_\mathrm {{ext}}}$ is the external work generated by the force applied with the indenter, ${U_\mathrm{S}}$ is the strain energy in the material, ${U_\mathrm{f}}$ is the energy dissipated due to friction, and ${U_\mathrm{G}}$ is the energy spent to cut the tissue. The kinematic energy is usually neglected in the description, provided that the insertion is performed at low rates. When the needle is itself deformable, the strain energy density should also include the elastic strain energy of the needle $U_\mathrm{SN}$, which to a first approximation can be correlated with the difference between the imposed displacement $D_\mathrm{e}$ and the effective vertical displacement at the tip *D*. If the needle is much stiffer than the sample, we can assume $D \simeq D_\mathrm {e}$ so that $U_\mathrm{SN} \simeq 0$. The general penetration of a needle consists of the following stages (Fig. 1):

1. *Initial indentation:* $$W_\mathrm {{ext}}=U_\mathrm{S}$$
  The external work is converted into elastic energy due to the deformation of the material under the action of the indenter (region 1). The stage ends once the strain energy accumulates to a critical value, at which a crack is initiated.
2. *Cut propagation until full penetration:* $W_\mathrm {{ext}}=U_\mathrm{S}+{U_\mathrm{f}} + U_\mathrm{G}$
  Once a crack is initiated, the external work is also consumed by frictional dissipation and work of fracture. Following initial rupture, there is a relaxation of the elastic strain energy (region 2a) and beginning of the cut propagation under increasing force (region 2b).
3. *Sliding after full penetration:* $W_\mathrm {{ext}}=U_\mathrm{f}$
  When the indenter has penetrated the material for the whole depth and breaks through the other end, there is another stage of relaxation, and the frictional resistance is the only remaining contribution (region 3).

### The programmable bevel-tip needle

**Fig. 2:**
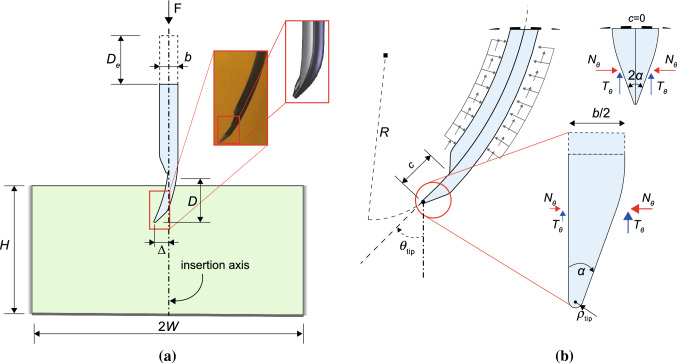
**a** Two-dimensional sketch of the programmable bevel-tip needle, inserted in a gelatine tissue phantom. **b** Enlarged views of the tip region, with the distribution of the tip forces. The components $N_\theta$ and $T_\theta$ are aligned with the local normal and tangential directions at the needle tip. The case of a symmetric tip ($c=0$), with balanced forces, is added for comparison

In the present study, we consider the flexible, programmable bevel-tip needle (PBN) designed at Imperial College, London (Burrows et al. 2013). Inspired by the ovopositor of certain wasps, this needle consists of four interlocked segments forming a thin and flexible shaft, a bevel tip and a programmable offset between the segments. The amount of offset between segments was shown to affect both steering magnitude and direction of the needle tip during the insertion process, in a predictable fashion. (See Ko et al. 2011 for 2D steering and Watts et al. 2019 for 3D.) A two-dimensional sketch of the needle and an enlarged view of the bevel tip are illustrated in Fig. 2a. Since we are investigating the in-plane steering, only two pairs of interlocked segments need to be considered: the relevant sizes are the needle diameter *b*, the bevel angle $\alpha$ and the programmable offset *c*.

During the insertion, PBNs follow curved paths, due to an asymmetric distribution of the resultant forces at the tip, as illustrated in Fig. 2b. In general, needles advance into the target material propagating a crack under a combination of mode I and mode II failure, with a tangential contribution coming from the sliding forces due to friction. When the tip is symmetric and sharp enough to avoid shearing the substrate, the opening mode I is predominant and the needle follows an approximately straight path (Shergold and Fleck 2005). Looking at PBNs in 2D, the tip deflection $\varDelta$ and the radius of curvature *R* depend on the amount of unsupported length associated with the offset *c* between the two segment pairs.

### The fracture process in needle penetration

When a needle is pushed through soft materials under an external force, a cut or crack is formed at the point of failure in the target substrate. If the material behaves as an elastic solid, the well-known concept of the strain energy release rate *G* provides a flexible approach to describe the process of energy accumulation leading to fracture. However, differently from standard fracture mechanics problems, needle penetration requires material failure to be captured in a way that enables tool–tissue interactions, taking into account not only the properties of the substrate but also the needle geometry.

Among the available numerical methods to describe failure and crack propagation, the cohesive zone model (CZM) has the capability to account for the nonlinear behaviour of soft materials and allows the inclusion of the contact constraints imposed by the penetration of the needle. In two-dimensional implementations of CZM, the fracture process zone is lumped into a line ahead of the crack tip, and the crack surfaces interaction is defined through a stress–displacement relationship describing the damaging mechanisms taking place close to the crack tip. The length of the cohesive process zone $L_\mathrm {coh}$ is proportional to the Young modulus *E*, the fracture energy $G_{\rm c}$ and the tensile strength $\sigma _\mathrm {max}$ (Hillerborg et al. 1976) according to:

2 $$\begin{aligned} L_\mathrm {coh}\sim \frac{EG_{\rm c}}{\sigma ^2_\mathrm {max}} \end{aligned}$$

**Fig. 3:**
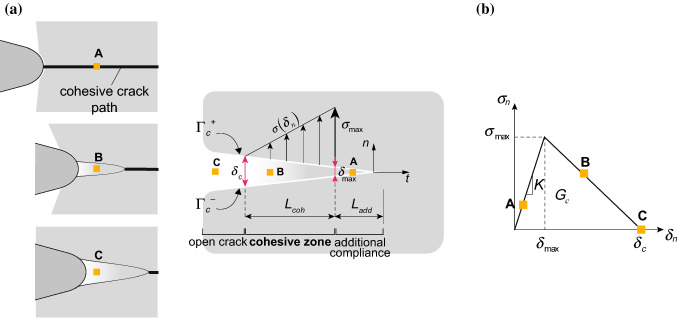
**a** The cohesive zone model concept (for better clarity, the normal behaviour is illustrated). A single point on the crack line is shown in three consecutive stages of insertion. Along the process zone length $L_\mathrm {coh}$, the cohesive stress $\sigma$ decreases from $\sigma _\mathrm {max}$ to zero, with the crack opening displacement $\delta _n$ rising from $\delta _{\mathrm {max}}$ to $\delta _{\rm c}$, at which the crack is fully open. The length $L_\mathrm {add}$ corresponds to an additional compliance introduced by the elastic part of the cohesive law. **b** Bilinear cohesive stress–displacement relationship for quasi-brittle materials, which is assumed equal in both normal and tangential directions

The stress–displacement law relates the traction $\sigma _j$ to the relative displacement $\delta _j$ between the upper ($\varGamma _{\rm c}^+$) and the lower ($\varGamma _{\rm c}^-$) crack surface (Fig. 3a):

3 $$\begin{aligned} \sigma _j =\sigma (\delta _j) \qquad \text {with} \qquad \delta _j=u_j^+-u_j^- \qquad j=t,n, \end{aligned}$$

where *t* and *n* denote the tangential and normal directions in a local reference system centred at the equivalent crack tip. Notice that in this work with equivalent crack we indicate the whole traction-free region and the cohesive part up to the point where $\delta _j\rightarrow 0$. Once the stress reaches the tensile strength $\sigma _\mathrm {max}$, known as the cohesive stress of the material, the crack initiates and then evolves to complete failure when the relative displacement equals the critical value $\delta _{\rm c}$.

Depending on the shape of the stress–displacement relationship, different types of failure can be described: for quasi-brittle materials, a possible choice is given by the bilinear curve (Zhang and Paulino 2005), where the onset of damage is followed by a linear softening down to complete failure (Fig. 3b). Notice that numerical implementation of the cohesive model requires a finite stiffness $K_{ij}$ prior to the onset of cracking, thus giving rise to elastic deformations and introducing an additional term $L_\mathrm {add}$ to the process zone length. As a consequence, the maximum cohesive stress is not attained at the equivalent crack tip (where $\delta _n=0$) but at the point corresponding to $\delta _{\mathrm {max}}$ (Fig. 3a). For a detailed explanation on the numerical implementation of CZMs, the reader is referred to the work by de Borst (2003).

The stress–displacement relationship of the bilinear model is written as:

4 $$\begin{aligned} {\sigma _i}= {\left\{ \begin{array}{ll} {K_ {ij} {\delta _j}} &\quad {{\delta _j} \le {\delta _{\max }}} \\ {\sigma _\mathrm {max}\frac{(\delta _{\rm c} - \delta _j)}{(\delta _{\rm c} - \delta _\mathrm {max})}} &\quad \delta _\mathrm {max}<\delta _j \le \delta _{\rm c}\\ 0 &\quad \delta _j > \delta _{\rm c} \end{array}\right. }. \end{aligned}$$

In this work, we have assumed the same cohesive relationship and cohesive energy for both the normal and the tangential direction ($K_ {ij} =K$ for $i=j$), without any coupling between the two ($K_ {ij} =0$ for $i\ne j$). We recall that the cohesive energy is related to the fracture energy $G_{\rm c}$ by the following relationship:

5 $$\begin{aligned} G_{\rm c}=\frac{1}{2} \sigma _\mathrm {max} \delta _{\rm c}. \end{aligned}$$

The cohesive model requires a careful choice of the parameters; in particular, one or both the fracture energy $G_{\rm c}$ and the cohesive stress $\sigma _\mathrm {max}$ should be obtained from experiments. Unfortunately, measuring the fracture energy of soft materials is not an easy task, due to the large strains preceding failure and the dependence on the strain rate. In the present study, we have followed an approach based on wire cutting, which relates the fracture toughness to the steady-state cutting force obtained from tests with wires having various diameters (Gamonpilas et al. 2009).

Also fundamental is setting the two relevant displacements $\delta _{\mathrm {max}}$ and $\delta_{\rm c}$, which need to be constrained to the geometry of the indenter, in order to perform a physically realistic simulation of the cutting process. A viable approach is to tune these parameters so that the simulated penetration force–displacement curve matches the experimental one (Oldfield et al. 2013a). In general, the critical displacement $\delta_{\rm c}$ is limited by the maximum diameter *b* of the needle: if this condition is not fulfilled, one would simulate the unrealistic situation of penetrating into the material without achieving a complete cut. The value of $\delta _{\mathrm {max}}$, although not envisaged in the original rigid plastic formulation of CZMs, has important consequences on the simulated fracture process. More specifically, large gaps between $\delta _{\mathrm {max}}$ and $\delta_{\rm c}$ imply that the material has a relevant supply of fracture resistance following damage initiation, before it is completely separated. In this work, we have assumed $\delta _{\mathrm {max}}$ to be equal to the tip diameter of the needle, $2\rho _\mathrm {tip}$, while we have adjusted $\delta_{\rm c}$ to be compatible with the limiting diameter *b*. This choice allows us to correlate the cohesive law with a parameter which gives a measure of the sharpness of the needle tip. In a generic cutting process, the role played by the tip radius is somewhat analogous to the so-called critical crack-tip opening displacement in standard fracture mechanics, with respect to the condition of crack propagation (Dunn et al. 2007). Indeed, several authors have shown that crack propagation during penetration is highly influenced by the tool sharpness, in terms of the resulting force, the shape of the crack and the finish of the separated surfaces (Shergold and Fleck 2005; McCarthy et al. 2007; Terzano et al. 2018).

## An iterative algorithm for needle penetration

### Description of the algorithm

**Fig. 4:**
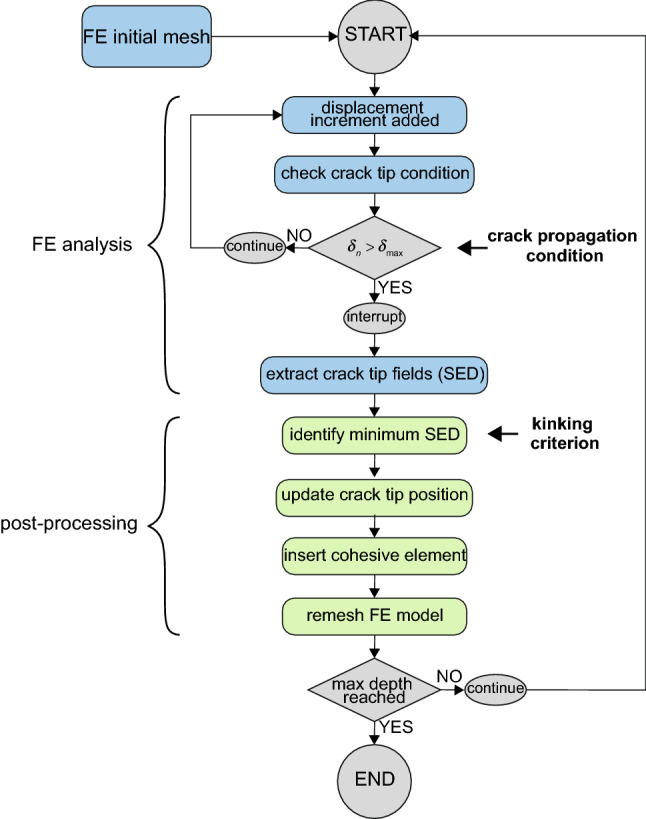
Flow chart showing the key steps of the penetration algorithm. Each iteration is divided in the two main stages of FE analysis and post-processing, and is repeated until the desired depth is achieved

In this section, we present the algorithm designed to simulate the penetration of a PBN into a soft elastic material. As the penetration path is not predetermined, an iterative method needed to be used. The iterative algorithm consists of two main parts: (i) the FE analysis and (ii) a post-processing step. The complete formulation is summarised in Fig. 4.

Part (i) is carried out with the commercial software SIMULIA Abaqus 2017 (Dassault Systèmes), using the implicit static solver and accounting for nonlinear geometry. Each iteration describes the penetration of the needle from the initial configuration ($D_\mathrm{e}=0$) to a certain displacement $D_\mathrm{e}^{(i)}$ (Fig. 2a). At this point, the FE analysis is interrupted because a further extension of the crack requires the definition of the direction of propagation. Part (ii) of the algorithm performs the post-processing and remeshing stages within a MATLAB environment. The local fields are employed to implement a certain fracture criterion. Subsequently, the mesh is updated in order to allow for the extension of the crack and to include new cohesive interfaces. We wish to point out that each iteration must start from the initial configuration ($D_\mathrm{e}=0$), because the insertion of new cohesive elements alters the stiffness of the model. Section 3.2 goes into the details of the mesh and the remeshing algorithm, while Sect. 3.3 presents the fracture propagation criterion and its numerical implementation.

### Initial FE mesh and remeshing algorithm

**Fig. 5:**
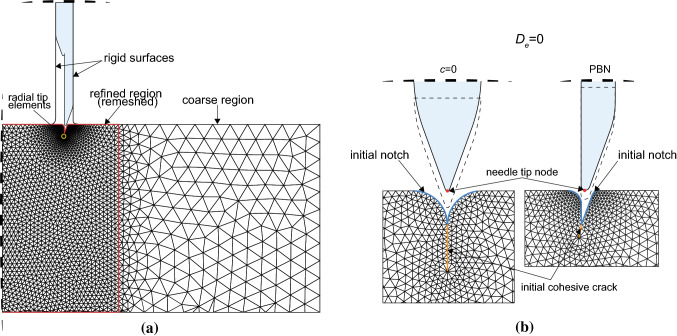
**a** General view of the initial configuration of the mesh (for clarity, the mesh of the needle is not shown). **b** Initial configuration in the case of a needle with a symmetric tip $c=0$ (left) and for the PBN (right). The initial notches in the substrate are highlighted in blue

A general view of the initial mesh is provided in Fig. 5a. Two different regions are identified in the substrate: an outer region and an inner region, where penetration is expected to take place. Both the needle and the substrate are modelled with plane strain linear triangular elements (CPE3 elements in Abaqus). The propagating crack is obtained by duplicating the nodes and inserting zero-thickness 4-node cohesive elements (COH2D4 elements in Abaqus), with a bilinear stress–displacement curve as defined in Sect 2.3. Element resolution is increased in the areas of the inner region, which are critical for contact interaction and the path of propagation. A special ring of elements (CPE3) is generated around the current equivalent crack tip, which controls the accuracy of the fracture criterion. The element size in this highly resolved zone is chosen in relation to a particular dimension of the needle. Considering the tip radius of the needle, the smallest element size *h* has a ratio $h/\rho _\mathrm {tip}=0.5$. The element size and mesh configuration have been tested for convergence with respect to more refined meshes, comparing the force–displacement curves resulting from the simulation. The mesh of the needle is also constructed with plain strain triangular elements, with a suitable refinement to ensure a good convergence during contact with the substrate.

Boundary conditions are applied to replicate a typical experimental set-up as closely as possible. The lateral surfaces of the substrate are pinned and the bottom nodes are also prevented from normal motion (restrained along the vertical direction). The needle is prevented from buckling by moving between two rigid vertical surfaces, which are frictionless, fixed and represent delivery through a trocar, which is common for flexible catheters. Contact constraints are enforced using a Lagrange multipliers method, with a frictional interaction between the needle and the substrate described by the Coulomb frictional law. Since the material of the substrate is highly deformable, it was necessary to introduce an initial notch to ease the convergence at the stage of indentation, as shown in Fig. 5b. The shape of this notch has been carefully designed with respect to the profile of the needle tip, and its effect was found to be negligible on the force–displacement profiles (except for the very initial part), and, more importantly, on the path of penetration. In the case of the needle with the symmetric tip ($c=0$), a line of ten cohesive elements—with greatly reduced strength in order to achieve initial convergence with large deformations—is predefined along a straight path. With asymmetric bevel tips, only one cohesive element, aligned with the bisector of the bevel angle $\alpha$, is inserted to facilitate the penetration.

The refined inner region of the substrate is remeshed at each iteration of the penetration algorithm, in order to accommodate the advancement of the needle. The remeshing stage is carried out in MATLAB, using the mesh generator DistMesh (Persson and Strang 2004). This choice was made as it is reasonably fast and allows accurate control of the mesh size, positioning and refinement around complex geometric features. At each mesh update, the ring of radial elements is shifted with the new equivalent crack tip and reoriented with the kinking angle, so that two element edges are always aligned with the current direction of propagation. In this way, crack propagation is not restrained to the element edges, but is effectively determined based on the results of the kinking criterion. Furthermore, since the analysis considers the nonlinear geometry effects, the crack direction is established on the current deformed configuration. The path of propagation, i.e. the coordinates of the nodes along the crack path, are stored at each iteration for the next one, so that at the last iteration the needle travels along a defined path down to the prescribed depth. In summary, it is only the last iteration that we consider for the results, with the propagation paths determined by the increments in the crack length that occurred in all the previous iterations.

### Crack propagation and kinking criterion

The CZM is a damage-based numerical model, in the sense that a crack is initiated once the stress within a cohesive element reaches a critical value. In this sense, a specific crack propagation criterion is not required, provided that the cohesive elements are inserted *a-priori* between the continuum elements. However, in our algorithm the propagation path is determined adaptively during the analysis, hence a fracture criterion is required to define the critical condition for propagation and the crack kinking angle.

**Fig. 6:**
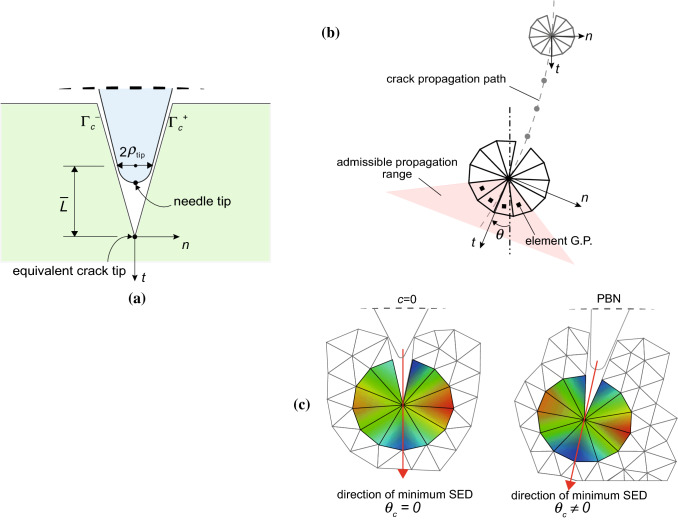
**a** Illustration of the critical condition for crack propagation: the needle tip is pushed forward until the critical CTOD at a distance ${\overline{L}}$ from the equivalent crack tip is reached. **b** Sketch of the crack propagation path, defined as the locus of the equivalent crack-tip positions during the iterations. In a generic point, *t* and *n* locate, respectively, the tangent and the normal directions to the propagation path. **c** Schematic showing the MSED kinking criterion, with the radial mesh around the equivalent crack tip in the deformed configuration. The coloured contours are related to the strain energy density, in the case of a needle with a symmetric tip $c=0$ (left) and for the PBN (right)

We introduce a fracture criterion based on the crack-tip opening displacement (CTOD), measured at a specified distance ${\overline{L}}$ behind the current equivalent crack tip. Such a distance has been defined taking into consideration the properties of the substrate and the geometric features of the needle (Fig. 6a):

6 $$\begin{aligned} {\overline{L}}=\frac{\rho _\mathrm {tip}}{\tan \alpha } \end{aligned}$$

where $\alpha$ is the bevel angle and $\rho _\mathrm {tip}$ is the tip radius. Assuming $2\rho _\mathrm {tip} = \delta _{\mathrm {max}}$, we find:

7 $$\begin{aligned} {\overline{L}}= \frac{\delta _{\mathrm {max}}}{2\tan \alpha } =\frac{\sigma _{\mathrm {max}}}{2K\tan \alpha } = L_\mathrm {add} \end{aligned}$$

Recalling that the CTOD is defined as $\delta _n=u_n^+ - u_n^-$ and assuming a critical CTOD equal to $\delta _\mathrm {max}$, the condition for crack propagation is formulated as:

8 $$\begin{aligned} \delta _n( {\overline{L}}=L_\mathrm {add})= \delta _{\mathrm {max}} \end{aligned}$$

In this way, we are indirectly relating the sharpness of the needle tip to the cohesive law of the material. As explained in Sect. 2.3, the use of a displacement-based fracture criterion enables us to consider the relevant influence of the tip radius and sharpness on the condition of crack propagation during penetration.

At this point, a criterion to determine the crack propagation direction needs to be defined. Several alternatives have been proposed in the literature (Bouchard et al. 2003; Sutula et al. 2018) that could be implemented in the algorithm. In this work, we have used the minimum strain energy density (MSED) criterion (Sih and Macdonald 1974), which has the advantage of adopting a global approach instead of a local field parameter (such as stress-based criteria). The kinking angle $\theta_{\rm c}$ is found considering the direction that minimises the strain energy density $U_\mathrm{S}$:

9 $$\begin{aligned} {\left\{ \begin{array}{ll} \left(\frac{{d}U_\mathrm{S}}{d\theta } \right)_{\theta =\theta_{\rm c}} &{}=0\\ \left(\frac{{d}^2U_\mathrm{S}}{d\theta ^2} \right)_{\theta =\theta_{\rm c}} &{}\ge 0 \end{array}\right. } \end{aligned}$$

where the second condition states that the point should be a point of minimum of the function $U_\mathrm{S}(\theta )$. In practice, from the ring of elements surrounding the current equivalent crack tip (Fig. 6b), strain energy densities are extracted at Gauss points and interpolated on an admissible angular range for propagation, based on the limit in pure shear loading (Sih 1974). The angle, within the admissible range, which corresponds to the local minimum of SED defines the crack propagation direction. Figure 6c illustrates the schematic contours of the SED in the case of a symmetric needle and for the PBN: in the latter, the SED distribution shows a minimum which is oriented at a certain angle with respect to the vertical direction of insertion.

**Fig. 7:**
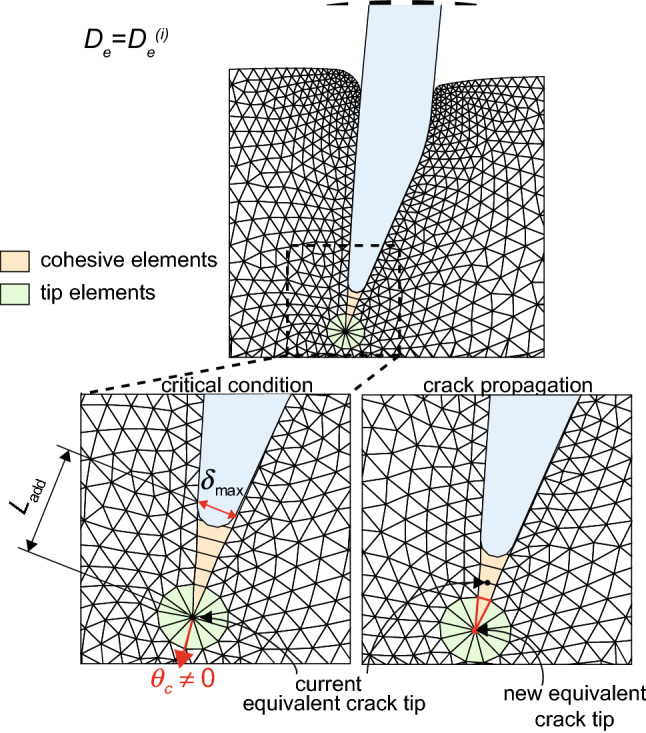
View of the deformed mesh during the penetration. The enlarged insets show the moment when the critical condition is reached, at a distance $L_\mathrm {add}$ from the current crack tip. At the same time, the kinking angle $\theta_{\rm c}$ is computed, according to the MSED criterion. The equivalent crack tip is propagated, and a new cohesive element is added (highlighted in red)

Figure 7 schematically shows how the whole fracture criterion is implemented in the iterative algorithm. Once the critical CTOD is reached at the distance $L_{\mathrm {add}}$ behind the current equivalent crack tip, the FE analysis is interrupted and the kinking angle is identified. At this point, the mesh needs to be updated with the insertion of an additional cohesive elements, deployed along the direction identified. The current equivalent crack tip is shifted a fixed distance, equal to the length of one element in the tip region, to the new position and the surrounding region is remeshed.

## Results and discussion

### Model parameters

The model used here is based on the experiments in Burrows et al. (2013), where two adjacent segments of the programmable bevel-tip needle were advanced equally to create a fixed offset that was maintained throughout the physical experiment. This configuration restricts needle steering to a single plane, and so a 2D plane strain simulation is used. The needle has an outer diameter of $\textit{b}=8\text { mm}$, a total bevel angle of $2\alpha = 20$°and tip radius equal to $\rho _\mathrm {tip}=0.5 \text { mm}$ (Fig. 2b). The offset *c* represents a programmable parameter which is changed between the simulations. The needle material is modelled as linear elastic, with a Poisson coefficient equal to $\nu =0.475$, whereas the Young's modulus $E_{\mathrm {needle}}$ is varied in the simulations, in the range 117–940 kPa. The substrate consists of a block of gelatine phantom, with a concentration of 10% by weight, having dimensions $\textit{2W}=235\text { mm}$ and $\textit{H}=245\text { mm}$. The gelatine material is also modelled as linear elastic, with Young’s modulus $E_{\mathrm {gel}}=14.8$ kPa and a Poisson coefficient $\nu =0.475$. It should be noted that although a linear elastic material description is used here, as in many other models available in the literature (Vigneron et al. 2011; Tai et al. 2013; Bui et al. 2019), the presence of large deformations is accounted for in the formulation. The mean value of the fracture energy $G_{\rm c}$ for the gelatine, obtained from wire-cutting tests at small strain rates, is assumed equal to 1.1 J/m^2^ (Forte et al. 2015). Contact interaction between the needle and the surrounding gelatine is represented by a coefficient of Coulomb’s friction equal to $f=0.3$, which agrees with the values obtained from cutting experiments in similar gelatine materials (Oldfield et al. 2013b).

A series of simulations were run on the PBN employing the algorithm described in Sect. 3. Separately, a symmetrical needle configuration, equivalent to a ‘zero offset’, was simulated to test whether a straight path could be achieved with the proposed procedure. Each simulation was run sufficiently long to reach a condition of steady state and obtain a stable curvature of the penetration path. Refer to the electronic supplementary material for animations of needle insertion, concerning the symmetric configuration (Online Resource1) and the PBN with offset $\textit{c}=47\text { mm}$ (Online Resource2). Please note that the animations are related to the last iteration of the algorithm (mesh adaptivity not explicitly shown), when the needle travels within the path obtained from all the previous iterations.

### Force–displacement curves

**Fig. 8:**
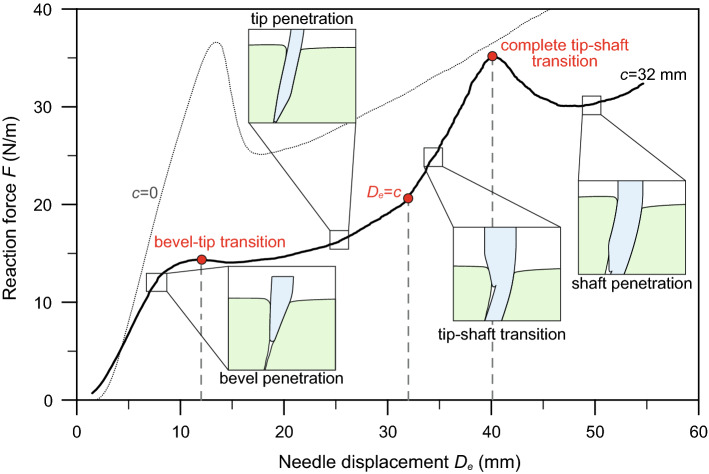
Force–displacement curve from PBN insertion simulation, with offset $c=32\mathrm {mm}$ and $E_\mathrm {needle}/E_\mathrm {gel}=16$. Insets on the plot are taken from the FE model. The thin dashed line results from the simulation with ‘zero offset’

The algorithm has proved able to reproduce different stages of the insertion of the programmable bevel-tip needle. The level of detail might be appreciated in Fig. 8, where a typical force–displacement curve obtained from the simulation is shown with the different stages of penetration. Compared to the overall characteristic shape of a symmetric needle insertion (thin dashed line in the figure) and the experimental results reported in Oldfield et al. (2013a), additional detailed features are captured by our numerical model. (Notice that the depth reached in this and the other simulations falls within the region 2b of Fig. 1.) After an initial stage, with almost linear increase in the reaction force, there is a first relaxation, corresponding to the completion of the bevel penetration. It should be noted that the presence of the initial notch in the gelatine mesh (Fig. 5b) is responsible for the lack of an initial sharp increase, which is usually present in the experimental curves and corresponds to the displacement at which a crack is initiated. After the first relaxation, the curve resumes a slightly increasing trend, up to the point corresponding to the end of tip penetration, i.e. when $D_\mathrm{e}=c$. At this point, the force rises rapidly to a maximum, corresponding to the complete tip–shaft transition, when the backward needle segment penetrates the material. Afterwards, a strong relaxation is followed by increasing force with the penetration of the needle shaft, which continues until full penetration (not reached in the numerical simulations). The second peak in the force, and the following relaxation, is absent in the symmetrical needle curve and is believed to be peculiar of the PBN insertion. Note that the force is expressed per unit of out-of-plane depth, since we have adopted a plane strain approximation. The values are consistent with those observed in experimental trials of needle insertion and cutting of similar gelatine materials (Oldfield et al. 2013a, b).

**Fig. 9:**
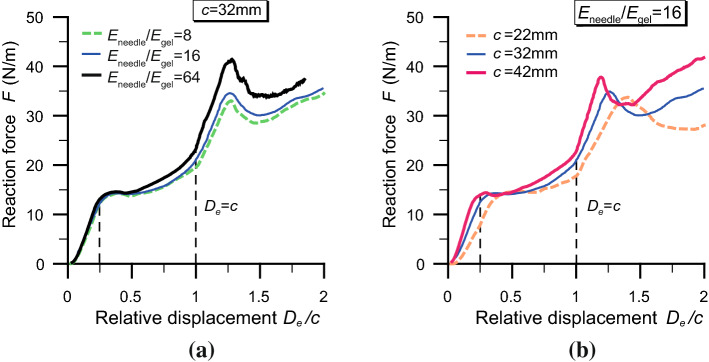
Force–displacement curves of the programmable bevel-tip needle. **a** Effect of the stiffness ratio $E_\mathrm {needle}/E_\mathrm {gel}$, at constant offset *c*. **b** Influence of the offset *c*, at constant stiffness. The vertical dashed lines mark the bevel-tip transition and the end of tip penetration ($D_\mathrm{e}=c$)

Force–displacement curves are known to be affected by various parameters, correlated with the needle geometry, the material elasticity and the characteristics of interaction. For instance, the penetration force has been shown to be sensitive to the bevel angle $\alpha$, with smaller bevel angles resulting in larger forces (Misra et al. 2008). While certainly worthy to be investigated, that parameter might not be the most relevant to consider when dealing with the peculiar steering mechanism of the PBN, which is controlled by the programmable offset *c*. For this reason, in this work we have run simulations changing the initial offset of the needle; additionally, we have also investigated the effects of the needle stiffness, keeping the offset fixed. Results of the analyses, in terms of the force profile during needle penetration, are reported in Fig. 9. The plots seem to suggest that changes in the needle–gelatine stiffness ratio have only limited effects on the maximum reaction force, with a 40 % increase following an eightfold growth of the needle Young’s modulus. Analogous behaviour has been observed in bevel-tip needles by other authors (Misra et al. 2008). The force is not influenced so much by changing the needle offset either, with the curves displaying only minor differences in their slopes with respect to the relative penetration. Both these parameters are expected to have a much larger impact on the needle trajectories.

### Needle trajectory and curvature

**Fig. 10:**
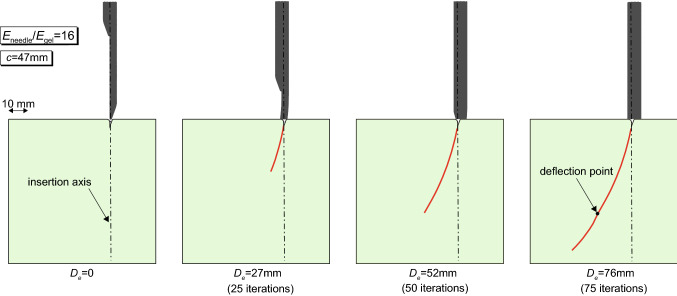
Trajectory of the PBN in the gelatine tissue phantom, for different needle displacements $D_\mathrm{e}$. The red line is the path taken by the needle tip node (the iterations required by the algorithm are specified in brackets)

An example of the typical trajectory followed by the needle in the simulations is illustrated in Fig. 10. The images are obtained from different iterations of the algorithm and overall show a smooth progression of the needle, which follows a curved path with an almost constant curvature. In the rightmost image, however, a deflection point is noticed, after which the path continues with a slightly increasing curvature, although smaller than the initial. This point corresponds to the completed transition from the needle tip to the thicker shaft, with an increase in bending stiffness of the needle, and is marked by the peak in the force–displacement curves (Figs. 8, 9). While such a deflection is difficult to capture in the experiments (Burrows et al. 2013), which either look at the needle insertion starting from positions already past the tip–shaft transition or do not have the resolution necessary to capture such effect, this shows that the full curvature evolution might not have stabilised (i.e. become constant) when the insertion is already progressed far into the soft material. This must be accounted for when comparisons with experiments are made.

**Fig. 11:**
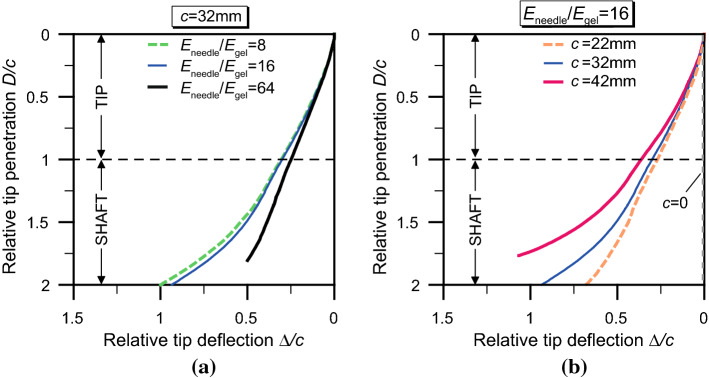
Penetration paths of the programmable bevel-tip needle. **a** Effect of the stiffness ratio $E_\mathrm {needle}/E_\mathrm {gel}$, at constant offset *c* and **b** influence of the offset *c*, at constant stiffness

The effect of the needle stiffness and the influence of the programmable offset *c* are again considered in Fig. 11, with respect to the needle penetration paths. These are defined in terms of the relative tip penetration *D*/*c* versus the horizontal deflection of the needle tip $\varDelta /c$. Figure 11b also illustrates the case of ‘zero offset’ between the needle segments, which as expected results in a straight path and provides a validation for the model. As observed by other authors, the needle deflection $\varDelta$ seems to be altered by both the needle elasticity (Misra et al. 2010) and the offset (Burrows et al. 2013), although it is not straightforward to quantify the exact dependence.

**Fig. 12:**
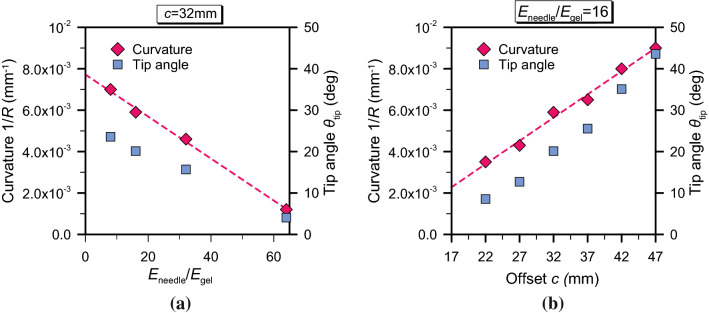
Variation of the tip angle $\theta _{\mathrm {tip}}$ and of the curvature 1/*R* with **a** the stiffness ratio $E_\mathrm {needle}/E_\mathrm {gel}$ and **b** the needle offset *c*. Both refer to a displacement equal to $D_\mathrm{e}=2c$

A better insight is gained by considering the needle curvature 1/*R* and the angle $\theta _\mathrm {tip}$ measured at the tip (Fig. 2b). The needle curvature is calculated from the path taken by the needle tip node, when the needle displacement is equal to $D_\mathrm{e}=2c$. Under the assumption of constant curvature along the whole trajectory, the radius of curvature *R* is determined as a parameter of the best fit circle, estimated using the hyper-circle algorithm (Chernov 2010). The angle $\theta _\mathrm {tip}$ is that formed by the local tangent direction at the needle tip and the vertical insertion axis and is computed from the first derivative of the hyper-circle at the specified depth. The results are reported in Fig. 12. The trends seem to suggest a linear dependence of both the curvature and the tip angle with the stiffness ratio and the needle offset. Specifically, Fig. 12b shows a linear increase in the curvature with growing offset. This provides good agreement with the experimental offset–curvature trends shown by Burrows et al. (2013), although the absolute values are lower.

**Fig. 13:**
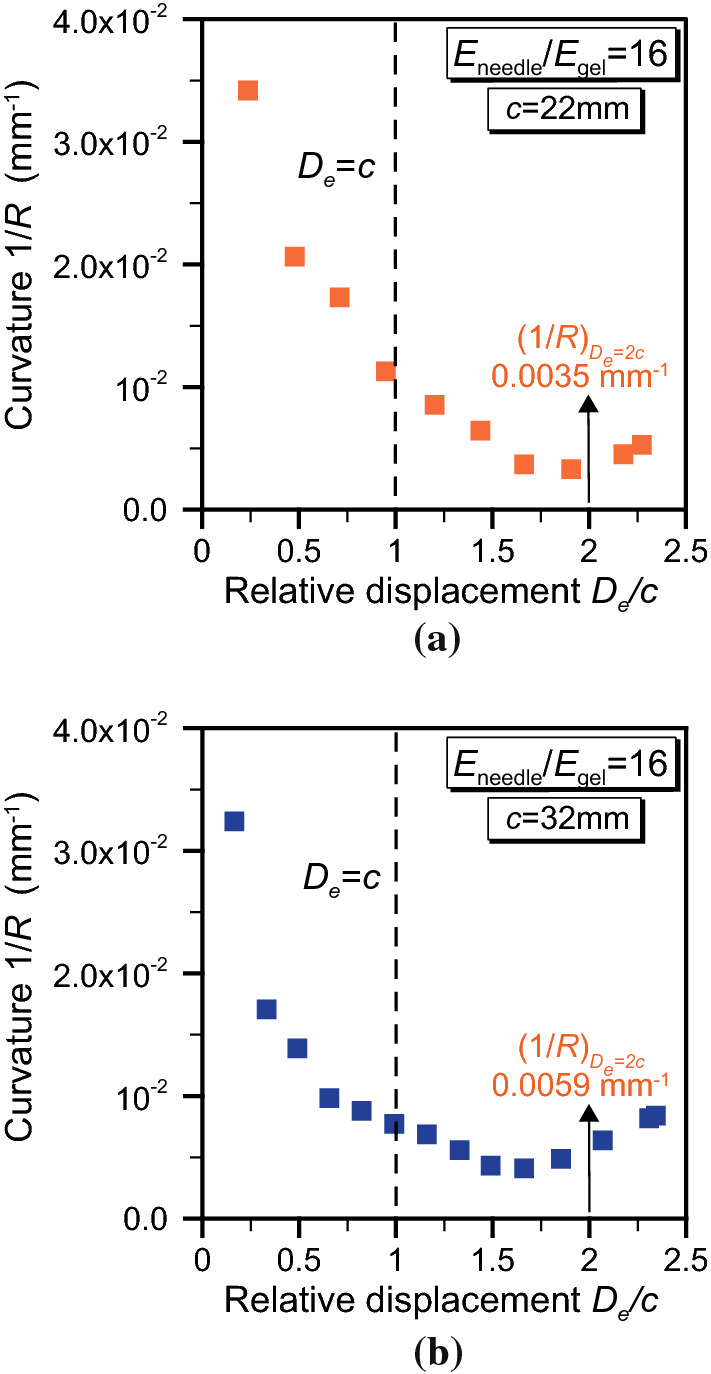
Variation of the curvature 1/*R* as a function of the relative displacement $D_\mathrm{e}/c$, for **a** $\textit{c}=22\text { mm}$ and **b** $\textit{c}=32\text { mm}$

The discrepancy might depend on various aspects. Firstly, the estimated curvature depends on the computation criterion and, in particular, on the depth at which it is calculated. This point might become evident if we explore the curvature variation with respect to the needle displacement (Fig. 13). To obtain this plot, we have removed the assumption of constant curvature and applied the hyper-circle fitting to increasing insertion depths, with a span of 5 mm. It is found that initially the curvature decreases sharply, until the needle has penetrated for a length approximately equal to the offset *c*. Then, the curvature appears to reach a minimum value at $D_\mathrm{e}\simeq 2c$ (compare the data reported in Fig. 12b), before starting a slightly increasing trend. The simulations are interrupted due to computational costs, as the increase in simulation time would have not provided any additional insight into describing the evolution of the penetration path, with attention focused on parametric study rather than deeper insertion. However, during experiments the needle is inserted further into the substrate, so it is reasonable to expect a larger curvature.

Other aspects to consider when comparing experiments and simulations are related to the characterisation of the tool–tissue interaction and, in particular, to the margin of error in quantifying the frictional behaviour. In this work, we have used Coulomb’s friction, although other authors have adopted a constant adhesive shear stress to model the frictional contact between soft materials and stiff tools (Atkins et al. 2004). The coefficient of friction has been chosen accordingly to similar experimental studies and has been shown to influence both the maximum force and the force–displacement gradient (Oldfield et al. 2013b), although a quantification of its effect on the curvature is still lacking. Frictional effects are expected to be lower when gelatine is replaced by other brain tissue surrogates, such as composite hydrogels (Leibinger et al. 2016). Lastly, the simplification introduced by the assumption of a planar insertion yields a stiffer behaviour with respect to the 3D case, since our 2D model is infinitely stiff in the out-of-plane direction. In general, a quantitative matching between experiments and simulation should be possible when all the parameters involved are carefully evaluated, but this is beyond the scope of the present paper.

The experimentally observed effects of a linear offset–curvature relationship (Burrows et al. 2013) provide a good point of validation for the modelling approach presented here. Physically, the behaviour seen in the model can be explained by the interaction of needle bending stiffness, substrate stiffness and the stress state around the needle tip during insertion. While not dramatically altering the overall curvature of the needle, kinks in the penetration path can be directly attributed to the change in bending stiffness as both segments of the needle interact with the tissue phantom substrate (Fig. 11). Other effects, such as the evolving curvature for different offsets (Fig. 13), are more complex. They are dependent on a more subtle coupling between the bending forces exerted on the offset bevel tip, the cut path in the substrate and the shaping influence this path has on the overall profile and curvature of the needle as it then passes through.

The nonlinearity caused by the relationship between offset, material stiffnesses and the pre-existing path is indicated when projecting the offset–curvature relationship in Fig. 12b to a zero offset. For the substantial offsets used here, and which would be typically necessary for curvatures with clinically significant benefit, the offset–curvature relationship is linear. However, at very small offsets the linearity breaks down and a more complex offset–curvature relationship would need to be established. It is clear that for no offset, forces around the needle tip are symmetric and a straight path is followed. For ‘small’ offsets, the more withdrawn bevel segment will have an influence on the distribution of stress around the tip. Similarly, the impact of increasing the stiffness of the needle at a fixed offset is highly intuitive yet also deteriorates at physically limiting situations. The linear relationship between needle stiffness and curvature (Fig. 12a) will break down if extrapolated to the assumption that a rigid needle results in no curvature. In practical terms, when there is a bevel present, even if the needle is rigid and undeformed, the shape of the bevel will still create a crack that is projected away from the insertion axis. On the other hand, when the needle stiffness becomes very low in relation to the forces at the tip, buckling is not preventable even with the practical assistance of trocar delivery. The modelling tool developed by the authors can certainly be used to shed light on all the aspects mentioned above and, therefore, to improve the design of PBNs.

## Conclusions

Needle steering during insertion in soft tissues is a complex phenomenon to be modelled with accuracy. Specifically, steering in programmable bevel-tip needles results from a combination of needle deflection, tool–tissue interaction forces and tissue deformation; as such, a high-resolution finite element model is believed to be the best choice for reliable simulations. In this work, we have proposed a detailed finite element model to reproduce the complete tool–tissue interaction occurring during penetration. A cohesive model has been used to describe the damage accumulation leading to fracture, which has been calibrated with parameters obtained from experimental tests. Cohesive elements are suitable to account for the large deformations occurring in the soft material, but need to be inserted between the continuum elements of the FE mesh to model the cutting process during penetration. For this purpose, an iterative algorithm has been devised, in which each iteration simulates the needle penetration from the initial configuration to a certain depth. A mixed-mode fracture criterion, based on the opening displacement and on the distribution of strain energy densities in the elements close to the crack tip, defines the critical condition for crack propagation. Based on such criterion, the iteration is interrupted to allow for an extension of the cohesive interface, followed by remeshing of the surrounding region.

The algorithm has been applied to deep insertion of a programmable bevel-tip needle in a gelatine tissue phantom. The simulation results show that our model can reproduce all the features of puncture and penetration of bevel-tip needles, providing a level of detail that has not been attained with other computational techniques. The parameters which are known to affect the penetration force and the steering capability of the needle have been carefully considered in this work. Specifically, we have shown how the needle stiffness reduces the curvature of the needle trajectory in the target material; moreover, we have also validated the experimental findings on the linear dependence of the curvature with the programmable offset. We wish to highlight that such results could not have been possible without a combination of a detailed model of the needle geometry with a physically based description of the failure process.

Notwithstanding the novelty of the proposed methodology, there are still some limitations that require careful consideration and provide inspiration for further developments. The computational cost inherent to the adaptive algorithm, together with some complexities in implementing the cohesive zone model, has for now prevented us from developing a full 3D model capable of delivering a comprehensive parametric study of practical use. This could be circumvented by looking at alternative algorithmic implementations and the use of hardware acceleration, e.g. taking inspiration from some of the interesting recent contribution reviewed in the Introduction of the paper. Our planar assumption, although largely adopted in similar works available in the literature, is an inexact approximation in most cases. Another simplification that we have introduced is related to the substrate material model. The approximation of a linear elastic behaviour is clearly a limitation, which might be acceptable for gelatine displaying a brittle behaviour, but certainly fails to describe adequately real tissues, such as the brain, or more complex tissue phantoms. Hyperelastic and viscoelastic models might offer a more realistic choice, and the advantage of the proposed approach is that it can be extended to nonlinear materials, since both the large deformations in the bulk and the nonlinear fracture process are adequately described by the computational approach adopted. It should be noted that viscoelastic materials are characterised by high dissipation in the regions of strain localisation, such as the crack-tip zone. A proper model which correctly describes fracture in these materials must consider the relationship between energy dissipation and the surface energy available for crack propagation.

From an applied perspective, the approach shown here proved to be suitable for systematically optimising material and geometric parameters for this needle steering mechanism. The potential for predicting the impact of different needle geometries, while reducing costly bespoke prototyping and the battery of experiments leading to design iterations, should not be underestimated. A predictive offset–curvature has further application in pre-operative path planning scenarios and ultimately by informing surgical simulators used to train clinicians in the deployment of devices.

## Electronic supplementary material

Below is the link to the electronic supplementary material.

Supplementary material 1 (mp4 24580 KB)

Supplementary material 2 (mp4 24585 KB)
